# Molecular characterization of a novel strain of *Bacillus halotolerans* protecting wheat from sheath blight disease caused by *Rhizoctonia solani Kühn*

**DOI:** 10.3389/fpls.2022.1019512

**Published:** 2022-10-17

**Authors:** Zhibin Feng, Mingzhi Xu, Jin Yang, Renhong Zhang, Zigui Geng, Tingting Mao, Yuting Sheng, Limin Wang, Juan Zhang, Hongxia Zhang

**Affiliations:** ^1^ College of Life Science, Ludong University, Yantai, China; ^2^ The Engineering Research Institute of Agriculture and Forestry, Ludong University, Yantai, China; ^3^ College of Agriculture, Ludong University, Yantai, China; ^4^ Key Laboratory of Molecular Module-Based Breeding of High Yield and Abiotic Resistant Plants in Universities of Shandong (Ludong University), Ludong University, Yantai, China; ^5^ Shandong Institute of Sericulture, Shandong Academy of Agricultural Sciences, Yantai, China

**Keywords:** *Bacillus halotolerans* LDFZ001, *Rhizoctonia solani Kühn*, antagonistic activity, sheath blight, wheat

## Abstract

*Rhizoctonia solani Kühn* naturally infects and causes Sheath blight disease in cereal crops such as wheat, rice and maize, leading to severe reduction in grain yield and quality. In this work, a new bacterial strain *Bacillus halotolerans* LDFZ001 showing efficient antagonistic activity against the pathogenic strain *Rhizoctonia solani Kühn* sh-1 was isolated. Antagonistic, phylogenetic and whole genome sequencing analyses demonstrate that *Bacillus halotolerans* LDFZ001 strongly suppressed the growth of *Rhizoctonia solani Kühn* sh-1, showed a close evolutionary relationship with *B. halotolerans* F41-3, and possessed a 3,965,118 bp circular chromosome. Bioinformatic analysis demonstrated that the genome of *Bacillus halotolerans* LDFZ001 contained ten secondary metabolite biosynthetic gene clusters (BGCs) encoding five non-ribosomal peptide synthases, two polyketide synthase, two terpene synthases and one bacteriocin synthase, and a new kijanimicin biosynthetic gene cluster which might be responsible for the biosynthesis of novel compounds. Gene-editing experiments revealed that functional expression of phosphopantetheinyl transferase (SFP) and major facilitator superfamily (MFS) transporter genes in *Bacillus halotolerans* LDFZ001 was essential for its antifungal activity against *R. solani Kühn* sh-1. Moreover, the existence of two identical chitosanases may also make contribution to the antipathogen activity of *Bacillus halotolerans* LDFZ001. Our findings will provide fundamental information for the identification and isolation of new sheath blight resistant genes and bacterial strains which have a great potential to be used for the production of bacterial control agents.

**Importance:**

A new *Bacillus halotolerans* strain *Bacillus halotolerans* LDFZ001 resistant to sheath blight in wheat is isolated. *Bacillus halotolerans* LDFZ001 harbors a new kijanimicin biosynthetic gene cluster, and the functional expression of *SFP* and *MFS* contribute to its antipathogen ability.

## Introduction

Wheat sheath blight has become one of worldwide disease causing severe yield loss crop plants (Ghosh et al., 2017). Necrotrophic fungus *Rhizoctonia solani Kühn*, which could produce host specific phytotoxins as pathogenicity or virulence factors, resulting in its widely spread and difficult to control, has been identified as the causal agent of sheath blight in many crops (Srivastava et al., 2016). Although Bacillus species have been widely used to control sheath blight, the antagonistic activity is still not high enough and needs to be improved (Abbas et al., 2019).

To date, various *Bacillus* species have been commercially used as pesticides, surfactants, and biological agents for flavor enhancing and nutrition supplementation, and about half of the commercially available bacterial control agents were originated from *Bacillus* species (Stein, 2005; Ongena and Jacques, 2008; Tareq et al., 2012; Tareq et al., 2014; Kim et al., 2017b; Pereira et al., 2019). The secondary metabolites, hydrolases and peptides, such as ribosomally synthesized and post-translationally modified peptides (RiPPs), nonribosomally synthesized peptides (NRPs), antitumor polyketides (PKs) and terpenes, which constitute a rich assortment of biologically active small molecules in the bio-control process, play a crucial role in plant pathogen inhibition (Tosato et al., 1997; Duitman et al., 1999; Tsuge et al., 2001; Luo et al., 2015b; Torres et al., 2015; Nair et al., 2016; Saggese et al., 2018). Based on the sequences of their genomes, gene clusters corresponding to different biological active molecules have been cloned, and their biological functions have been identified in different bacterial species (Gao et al., 2017; Jin et al., 2017).

To understand the functions of biological active molecules produced by microorganism for plant pathogen protection, complete genome sequencing has been taken as an efficient strategy (Chen et al., 2009b; He et al., 2013; Guo et al., 2015; Shaligram et al., 2016; Jin et al., 2017; Jadeja et al., 2019; Lu et al., 2019; Pereira et al., 2019). Compared with the genome sequence of *B. subtilis* 168, the first sequenced model organism of *Bacillus* species, different sequences responsible for cell wall and antibiotic synthesis have been identified, implying the functional difference of different genome sequences in *Bacillus* species (Guo et al., 2013; Sabaté and Audisio, 2013; Bóka et al., 2019). In the genome of *B. subtilis* 168, almost 4% of the whole genome was predicted to be responsible for the encoding of multifunctional enzymes involved in antibiotic biosynthesis (Kunst et al., 1997). By comparing the genome sequences between different microorganisms, some new gene clusters synthesizing novel antimicrobial products have been predicted (Chen et al., 2009a; Luo et al., 2015a; Mobegi et al., 2017).

Surfactins, the biosurfactant molecules widely identified in *Bacillus* species, have showed multiple bio-activities (Kim et al., 2017a; Li et al., 2021; Han et al., 2022). As a member of lipopeptide family, surfactins synergistically affected the bio-control effectivity of *Bacillus* species (Li et al., 2016; Kim et al., 2017b). Full genome sequence annotation analysis indicated that a surfactin operon, including *srfA*, *srfB*, *srfC*, and *srfD*, was responsible for the biosynthesis of surfactin (Nakano et al., 1991). Meanwhile, a genetic locus *sfp*, encoding a phosphopantetheine transferase, was also required for surfactin production (Wu et al., 2019). Although *B. subtilis* strain 168 possessed a complete *srf* operon, it was unable to produce surfactin due to a frameshift in the *SFP* gene, which resulted in the production of an inactive phosphopantetheine transferase. Integration of a functional *SFP* gene restored the ability of *B. subtilis* 168 to synthesize surfactin (Reuter et al., 1999; Wu et al., 2019).

In addition to *SFP* gene, other genes in *srf* operon, such as major facilitator superfamily (MFS) proteins, were also identified *via* genome sequence analysis. To date, MFS transporter was the largest transporter superfamily, including over 10,000 members divided into 74 families (Marger and Saier, 1993; Saier et al., 1999; Wang et al., 2020; Saier et al., 2021). MFS transporters could facilitate the transport of a variety of substrates, including ions, sugar phosphates, drugs, nucleosides, amino acids and peptides, across cytoplasmic and internal membranes (Saier and Paulsen, 2001; Lorca et al., 2007; Chen et al., 2008; Yen et al., 2010). Crystallographic structures of MFS members consisted of a typical 12 transmembrane segments and a unique intracellular four-helix domain (Madej and Kaback, 2013). Most MFS transporters in some bacteria transported specific substrates and were closely related with the immunological issues such as virus invasion and drug resistance (Manel et al., 2005).

Chitosanase could specifically catalyse the hydrolysis of the β-1,4-glycosidic linkage in chitosan, to produce chitosan oligosaccharides, the only natural alkaline amino oligosaccharides widely used in pharmaceutical, food and cosmetic industry (Park et al., 1999; Kurakake et al., 2000; Omumasaba et al., 2000; Yoon et al., 2002; Wang et al., 2008; Johnsen et al., 2010; Kang et al., 2012). Chitosanases also have a function in plant pathogen suppression (Zhao et al., 2011). In prokaryotes, chitosanase, whose target chitosan is not a constituent of the cells, is alleged to work as extracellular enzymes (de Araújo et al., 2016; Park et al., 1999; Liang et al., 2014). A gene cluster encoding chitosanase, which was able to prevent plant from the infection by *Plasmodiophora brassicae*, a common pathogen that causes clubroot disease, has been identified (Guo et al., 2013).

In the past years, a number of *Bacillus* strains have been isolated and the possible functions of some gene clusters in their genomes have been examined. However, *Bacillus* strain showing significantly protection of wheat from sheath blight disease caused by *Rhizoctonia solani Kühn* is still not identified. In the present study, a new *Bacillus* species with high antifungal activity and great potential for sheath blight protection in cereal crops was isolated, and its genome sequence and the possible genes responsible for the antifungal activity were investigated.

## Materials and methods

### Strains and culture conditions

*Bacillus halotolerans* LDFZ001 (*B. halotolerans* LDFZ001) was isolated from the sandy soil collected from the coastal zone of Yantai city, Shandong province, China, using serial dilution plating methods. Single colony was cultured on LB medium and stored at -80°C. The control strains *Bacillus subtillis* 168 (*B. subtillis* 168) and *Bacillus halotolerans* F41-3 (*B. halotolerans* F4103) were purchased from BioSciBio (Hangzhou, China). The pathogenic strain *Rhizoctonia solani Kühn* sh-1 is a collection in our lab. All the bacterial and fungus strains used in this study were listed in **Table S1**.

For the cultivation of *B. halotolerans* LDFZ001, *B. subtillis* 168 and *B. halotolerans* F41-3, nutrient broth (NB) liquid medium consisting of 3 g beef extract, 10 g peptone, 5 g NaCl, 2 g MgCl_2_ per liter was used. For the growth and preservation of the pathogenic strain *Rhizoctonia solani Kühn* sh-1, potato dextrose agar (PDA) medium consisting of 200 g Patato Destrose Agar (Coolaber, Beijing, China), 20 g sucrose, 20 g agar, was used.

The cloning plasmid pEASY-T1 was purchased from TransGen Biotech (Beijing, China). The expression plasmid pET28a (+) and the host strain *E. coli* BL21 (DE3) were purchased from Novagen (Shanghai, China). The plasmid pJOE8999 for gene editing with CRISPR-Cas 9 system is a collection in our lab. All the materials for gene cloning and protein purification were purchased from TaKaRa Biotechnology (Dalian, China). HisTrap HP and HiTrap Desalting were purchased from GE Healthcare (München, Germany). Chitosan for enzyme assay was obtained from Sigma-Aldrich (St. Louis, USA). Other chemicals were purchased from Sangon Biotech (Shanghai, China).

### Morphology observation and identification of bacterial strain

The morphology of purified strain *B. halotolerans* LDFZ001 was observed with light microscope (Olympus BX41, Japan) at a magnification of × 1000. Genomic DNA, isolated from the purified bacterial strain was used as template to amplify the 16S rRNA gene. The corresponding sequence was applied to phylogenetic analysis using MEGA 7.0 (Kumar et al., 2018).

### Antipathogen activity analysis

For antifungal activity analysis, pathogenic microbe strain *R. solani Kühn* sh-1 was inoculated at the center of PDA medium plate and cultured at 28°C alone or with *B. halotolerans* LDFZ001, *Bacillus subtillis* 168 or *B. halotolerans* F41-3 for three days, which was inoculated as a scratch line under the *R. solani Kühn* sh-1 inoculation spot on each plate. Then, the inhibition rate (IR) of *B. halotolerans* LDFZ001, *B. subtillis* 168 and *B. halotolerans* F41-3 against *R. solani Kühn* sh-1 was evaluated as described previously (Chen et al., 2019).

For the inhibition efficiency analysis of *B. halotolerans* LDFZ001, *B. subtillis* 168 and *B. halotolerans* F41-3 on *Rhizoctonia solani Kühn* sh-1 caused wheat sheath blight, *Rhizoctonia solani Kühn* sh-1 cultured on NB solid medium at 28°C for 24 were collected by centrifugation, washed two times with 0.05 M sodium phosphate buffer (pH7.2), and re-suspended with it to a final concentration of 2×10^8^ CFU/mL. One-week-old wheat seedlings germinated on filter paper soaked with sterile water were sprayed with 10 mL of the prepared bacterial solution. After 24 h, a 5 mm agar block of *Rhizoctonia solani Kühn* sh-1 grown on NB solid medium were inoculated to the middle of hypocotyls. After incubated at 25°C for 5 days, the phenotypes of seedlings were observed. The disease severity was graded according to the previous reported standard (Chen et al., 2019). The disease incidence rate (DIR) was generated using the following formula:


$$DIR(\%)=\frac{n}{N}\times 100$$

N represents the total number of investigated plants and n is the number of infected plants (Li et al., 2019). In our study, 30 plants were selected to conserve and investigate the DIR for each treatment.

### Crude lipopeptide preparation and determination

Cells of *B. halotolerans* LDFZ001, *B. subtillis* 168 and *B. halotolerans* F41-3 were cultivated in NB liquid medium at 28°C for 48 h. The concentrations of the fermentation broths were calibrated to OD_600 =_ 1.0. Then 100mL of the calibrated fermentation broths from these three bacteria were separately centrifuged at 8000 rpm/min for 10min to remove the bacteria. The supernatants were modified to pH=2 by 6mol/L hydrochloric acid and placed in 4°C refrigerator for more than 12h. After centrifugation at 8000 rpm/min for 10 min, the precipitate was collected. The crude extract was washed twice and dissolved in 100μ of methanol for HPLC analysis and antifungal activity assay. For HPLC analysis, crude extracts were filtered with a 0.22 μm membrane filter. Mobile phase was a mixture of acetonitrile and H_2_O (85:15, v/v). The flow rate was 1.00 mL/min. The injection volume was 10μL. The temperature was set at 28°C. Agilent C18 (250×4.6mm, 5µm) column was use and the detection wavelength was 210nm. Lipopeptides were analyzed using an Ultra high liquid chromatography system with a high resolution mass spectrometer (MS) as described previously (Chen et al., 2018).

The Oxford cup method was performed to analyze the antifungal activity of crude lipopeptides. *Rhizoctonia solani Kühn* sh-1 cultured on NB liquid medium at 28°C for 24 h were spread evenly on PDA solid medium. Two days later, four agar blocks (3mm×3mm) with the *Rhizoctonia solani Kühn* sh-1 were cut and place on a PDA solid medium plate, in the center of which a sterilized Oxford cup was placed. Using ddH_2_O as a control, 200μL of crude lipopeptide was dripped into the Oxford cup, which was taken away after 12h. The plates containing crude lipopeptide and agar blocks of the *Rhizoctonia solani Kühn* sh-1 were placed in 28°C for 72h. The inhibition zones were observed, photographed and measured.

### Genome sequencing and assembly

High-quality genomic DNA isolated from *B. halotolerans* LDFZ001 with Wizard^®^ Genomic DNA Purification Kit (Promega) according to manufacturer’s protocol was quantified with TBS-380 fluorometer (Turner BioSystems Inc., Sunnyvale, CA), and applied to a combination of PacBio RS II Single MoleculeReal Time (SMRT) and Illumina sequencing platforms for sequencing. Then, data generated were analyzed using I-Sanger Cloud Platform (www.i-sanger.com) from Shanghai Majorbio (Shanghai, China).

### Gene prediction and annotation

CDS, tRNA and rRNA were respectively predicted with Glimmer (version 3.02, http://cbcb.umd.edu/software/glimmer/), tRNA-scan-SE (version 1.23, http://lowelab.ucsc.edu/tRNAs.can-SE) and Barrnap (version 1.2, http://www.cbs.dtu.dk/services/RNAmmer/). The predicted CDSs were annotated from the non-redundant (NR) NCBI database, Swiss-Prot (http://uniprot.org), Pfam, GO, COG (http://www.ncbi.nlm.nih.gov/COG) and KEGG (http://www.genome.jp/kegg/) database using sequence alignment tools BLAST, Diamond and HMMER. Briefly, each set of query proteins was aligned with the databases, and annotations of best-matched subjects (e-value< 10^-5^) were obtained for gene annotation.

Secondary metabolite gene clusters were predicted with the online tools NP searcher (http://dna.sherman.lsi.umich.edu/) and antiSMASH (http://antismash.secondarymetabolites.org/). The genome of *B. halotolerans* LDFZ001 in a circular format was obtained using Circos.

### Plasmid construction and transformation

Deletion of the gene’s chromosomal region was performed as described previously (Altenbuchner, 2016). PCR fragments from the upward and downward regions of the gene were inserted into the downstream of T7 promoter in plasmid pJOE8999. To generate plasmids pJOEsfp and pJOEmfs for *B. halotolerans* LDZF001 transformation, the sgDNAs of the relative genes to be deleted based on the database from the internet (http://crispor.tefor.net/crispor.py) were separately inserted into these resultant plasmids *via* homologous recombination (Anagnostopoulos and Spizizen, 1961; Ge et al., 2015; Altenbuchner, 2016). Using conventional two-step procedure, the strain was cultured in a minimal medium (Peptone 10g/L, Yeast extract 5g/L, NaCl 10g/L, Sorbitol 91g/L, pH7.0) to an of OD_600_ value of 0.8 at 30°C. After harvested *via* centrifugation at 4 °C, the cell pellet was re-suspended in ETM buffer (Sorbitol 91g/L, Mannitol 91g/L, 10% Glycerol (v/v), pH 7.0) for electroporation (1800v, 200Ω, 25μF). Competent cells containing pJOEsfp or pJOEmfs plasmid were recovered in RM medium (Peptone 10g/L, Yeast extract 5g/L, NaCl 10g/L, Sorbitol 91g/L, Mannitol 69g/L, pH7.0) and spread on the RM solid plates with supplemented with 40 mg/L kanamycin. The transformant colonies were tested with PCR after the plasmid was cured by being cultured at 37°C for 12-16h.

For heterologous expression of Csn-gene1288 and Csn-gene2656, the two chitosanase genes from *B. halotolerans* LDFZ001 were cloned into pET28a(+) to generate the recombinant plasmids pET28a-Csn1288 and pET28a-Csn2656 *via* homologous recombination (Ge et al., 2015). The resultant constructs were subsequently transformed into *E. coli* BL21 (DE3). After induced with IPTG, the recombinant proteins were purified *via* immobilized metal affinity chromatography. Chitosanase activity was measured as describe previously (Kurakake et al., 2000; Kang et al., 2012). The reaction was performed at 50°C for 15 min in sodium acetate buffer (pH5.4), followed by the determination of reducing sugar using DNS methods. The optimal condition assays were performed by measuring the activity in sodium acetate buffer (pH 3.6-7.0) at specific temperatures ranging from 30°C to 75°C. All the primer sequences used in this study were shown in **Table S2**.

### Statistical analysis

For the antifungal assay, three replicates were performed. Student’s t-test with IBM SPSS Statistics 21 was performed to generate every P value (*P< 0.05).

## Results

### Isolation and identification of *Bacillus halotolerans* LDFZ001

**Figure 1:**
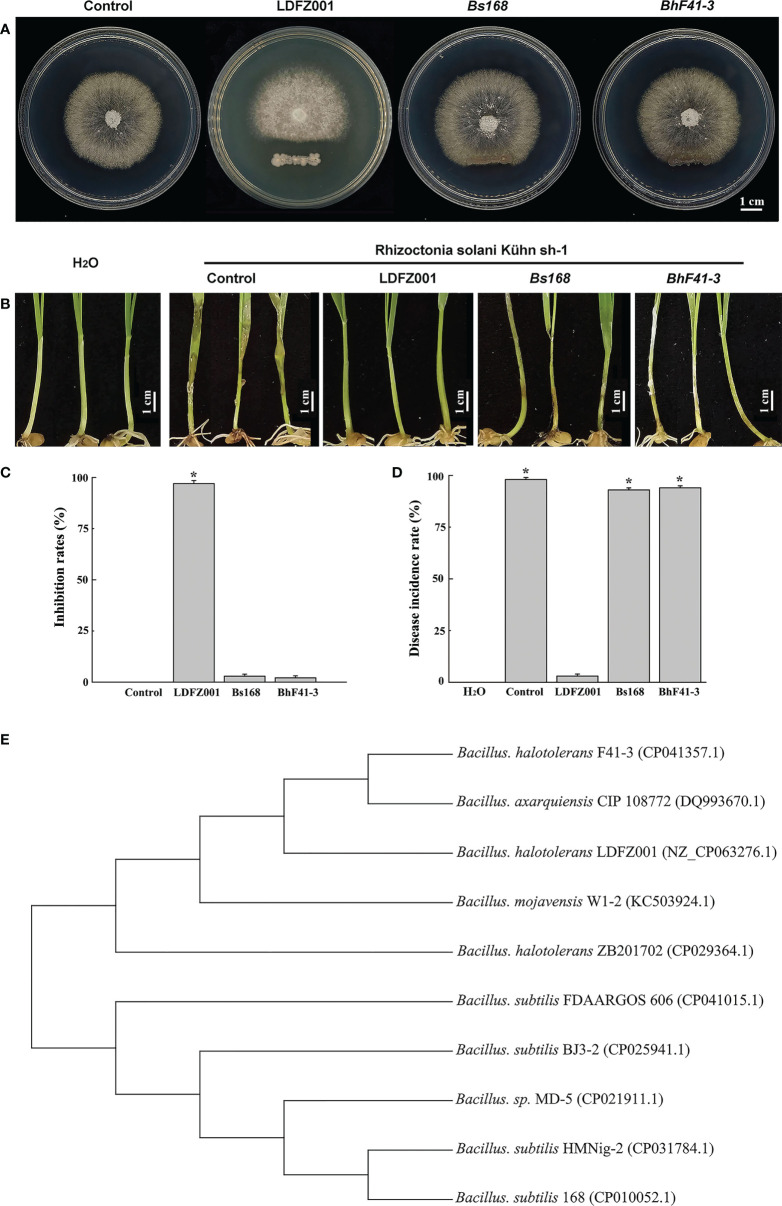
Antipathogen activity and phylogenetic relationship analyses. **(A)** Comparison of the antipathogen activity of *B halotolerans* LDFZ001 (LDFZ001) with its relative strains *Bacillus subtillis* 168 (*Bs168*) and *Bacillus halotolerans* F41-3 (*BhF41-3*) against *R. solani Kühn sh-1*, a pathogenic fungus strain cased sheath blight disease in crop plants. *R. solani Kühn sh-1* inoculated at the center of PDA medium plate was cultured three days at 28°C alone or co-cultured with LDFZ001, *Bs168* or *BhF41-3*, which was inoculated as a scratch line under the *R. solani Kühn sh-1* inoculation spot on each plate. **(B)** Susceptibility analysis of wheat seedlings pretreated with LDFZ001, *Bs168* and *BhF41-3* to *R. solani Kühn* Sh-1.The sodium phosphate buffer was used as negative control (control). Wheat seedlings without any treatment were shown in the left as a control, too. **(C)** Inhibition rates (IRs) of *B halotolerans* LDFZ001, *B subtillis* 168 and *B halotolerans* F41-3 against *R. solani Kühn* sh-1 in image **(A)**. **(D)** Disease incidence rates (DIRs) in image **(B)**. Values are the mean ± SD from three independent experiments (n = 30). *P< 0.05. **(E)** Phylogenetic tree of *B halotolerans* LDFZ001 and other related taxa was generated based on 16S rRNA sequence. MEGA 7 was used to align the sequences.

To identify new antagonistic strain against fungal pathogen, we isolated a total number of 26 bacterial clones from the sandy soil in the coastal zone of Yantai, Shandong Province of China, and assessed their antagonistic activities against the pathogenic strain *Rhizoctonia solani Kühn* sh-1 which caused sheath blight disease in most crop plants. We found that one clone, numbered as LDFZ001, displayed very strong *in vitro* antagonistic activity against *Rhizoctonia solani Kühn* sh-1. Therefore, LDFZ001 was chosen for further studies. It is well known that *Bacillus* species possessed antagonistic activity against various pathogenic fungi. Therefore, two *Bacillus* strains, *B. subtillis* 168 and *B. halotolerans* F41-3, along with LDFZ001, were respectively co-cultivated with *Rhizoctonia solani Kühn* sh-1 on PDA medium. LDFZ001 effectively suppressed the radical growth of *Rhizoctonia solani Kühn* Sh-1, whereas *B. subtillis* 168 and *B. halotolerans* F41-3 did not. LDFZ001 exhibited a 98.8%, whereas *B. subtillis* 168 and *B. halotolerans* F41-3 only showed 2.3% and 1.4%, inhibition rates on the radical growth of *Rhizoctonia solani Kühn* Sh-1, respectively (**Figures 1A, C**).

Since *Rhizoctonia solani Kühn* Sh-1 can produce basidiospores, which cause the damping off and stem rot in wheat seedlings. We further compared the protective ability of *B. subtillis* 168, *B. halotolerans* F41-3 and LDFZ001 against wheat sheath blight on wheat seedlings caused by *R. solani Kühn* sh-1. We observed that one-week-old wheat seedlings pretreated with LDFZ001 successfully protected the occurrence of wheat sheath blight, but seedlings pretreated with *B. subtillis* 168 or *B. halotolerans* F41-3 did not. The seedlings were susceptible to *R. solani Kühn* Sh-1 and showed almost the same wheat sheath blight phenotype as did the negative control seedlings treated with sodium phosphate buffer, accompanied with a very high disease incidence rate (**Figures 1B, D**).

To determine the properties of LDFZ001, we performed morphological observation. LDFZ001 colonies cultured on LB medium plate formed approximate circles with creamy smooth surface and regular edge. Morphological analysis with light microscopy demonstrated that LDFZ001 cells were gram-positive and in rod shape (data not shown). To further determine the phylogenetic relationship of LDFZ001 with other bacterial strains, a neighbor-joining tree based on 16S rRNA sequence was constructed with MEGA 7.0. Compared with other *Bacillus* family members, LDFZ001 showed close evolutionary relationship with *B. halotolerans* F41-3 and *B. mojavensis* W1-2 (**Figure 1E**). Therefore, LDFZ001 was named as *Bacillus halotolerans* LDFZ001, and was deposited in the China General Microbiological Culture Collection Center with an accession number of CGMCC 7187.

### The anti-pathogen activity of *Bacillus halotolerans* LDFZ001 is associated with lipopeptide production

**Figure 2:**
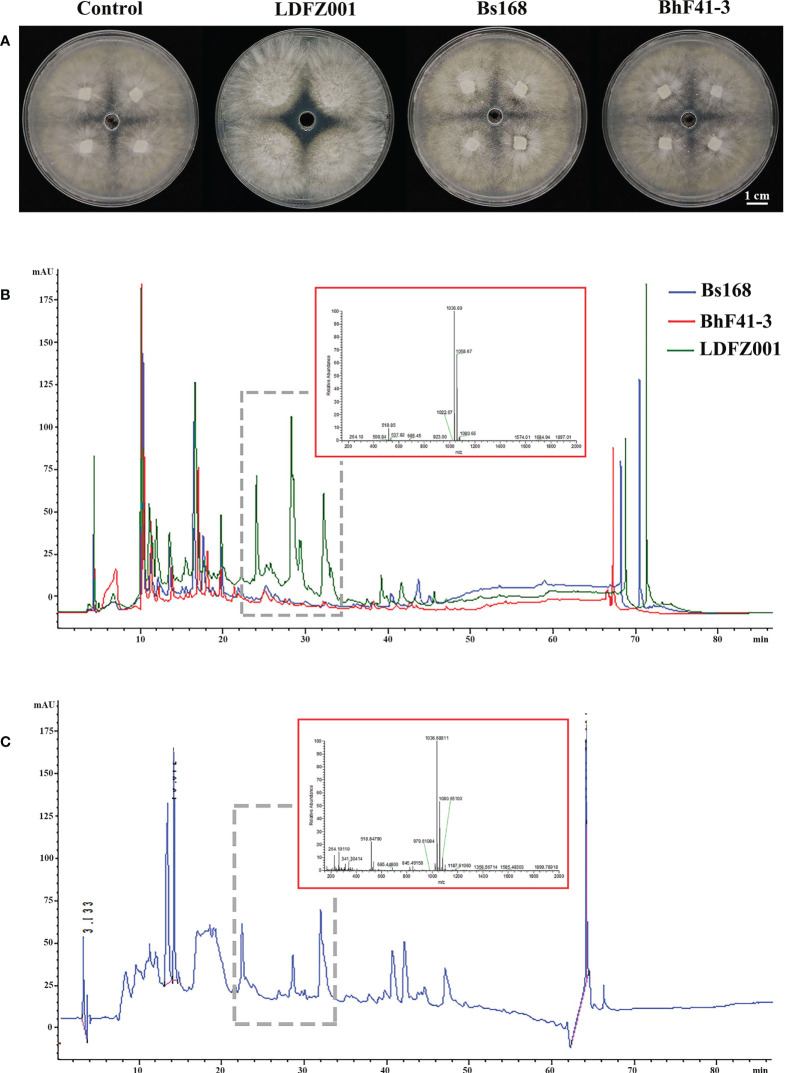
Lipopeptide extracts from *B halotolerans* LDFZ001 (LDFZ001), *Bacillus subtillis* 168 (*Bs168*) and *Bacillus halotolerans* F41-3 (*BhF41-3*) were used for antifungal activity and HPLC analyses. **(A)** Antifungal activity assays of putative lipopeptides against *R. solani Kühn* sh-1 **(B, C)** Profiling comparisons of HPLC and the extracted ion flow spectra of m/z 200–2000 from different strains.

Lipopeptides play a crucial role in the protection of plants from fungal pathogen attack. To understand whether lipopeptides also make a contribution to the antagonistic activity of *B. halotolerans* LDFZ001 against *Rhizoctonia solani Kühn* Sh-1, lipopeptide extracts from *B. subtillis* 168, *B. halotolerans* F41-3 and *B. halotolerans* LDFZ001 were separately isolated. Similarly, lipopeptide extract from *B. halotolerans* LDFZ001 significantly prohibited the growth of *Rhizoctonia solani Kühn* Sh-1. A clear inhibition zone of about 6.11 cm^2^ against *Rhizoctonia solani Kühn* sh-1 was observed. However, lipopeptide extracts from *B. subtillis* 168 and *B. halotolerans* F41-3 did not. The growth of *Rhizoctonia solani Kühn* Sh-1 was about the same as that on the control plate (**Figure 2A**). We then carried out HPLC assays with the lipopeptide extracts from *B. subtillis* 168, *B. halotolerans* F41-3 and *B. halotolerans* LDFZ001, a serial of distinct peaks in the profile of *B. halotolerans* LDFZ001, but not in the profiles of *B. subtillis* 168 and *B. halotolerans* F41-3, were observed (**Figures 2B, C**). Using UPLC-ESI-MS, these distinct peaks were determined as antifungal lipopeptide surfactin A (**Figures 2B, C**). Ions of m/z values 1022.67, 1036.69 and 1058.67 were also detected in previous reports (Roongsawang et al., 2002; Chen et al., 2019).

### High genome sequence identity is observed between *B. halotolerans* LDFZ001 and *B. halotolerans* F41-3

**Figure 3:**
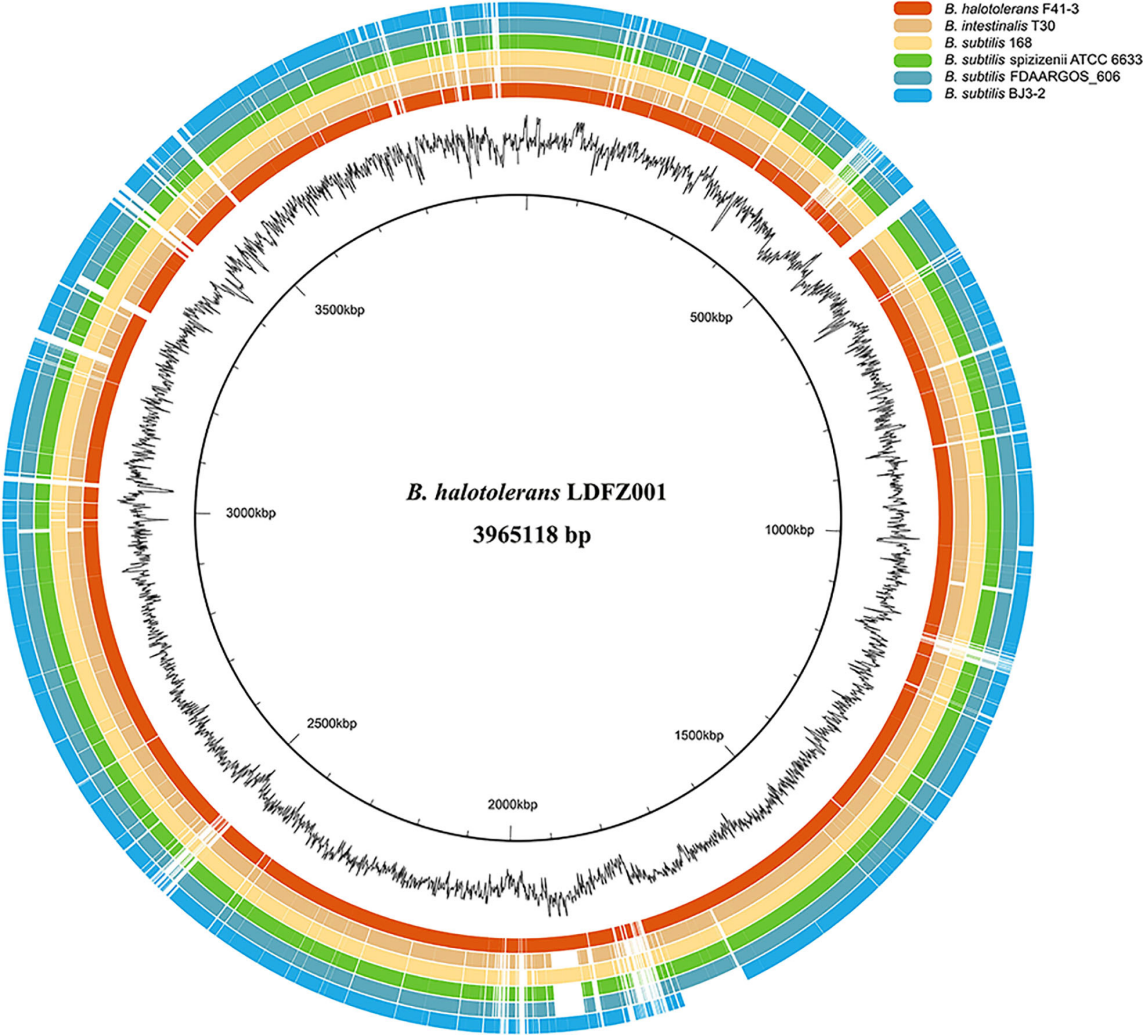
Genome comparison of *B halotolerans* LDFZ001 with six of its close *Bacillus* species. The innermost black circle represents the genome of *B halotolerans* LDFZ001 followed by its GC content and the *Bacillus* genomes.

To identify the responsible genes for the antifungal activity of *B. halotolerans* LDFZ001, we performed whole genome sequencing of *B. halotolerans* LDFZ001. The whole genome was assembled into a 3,965,118 bp circular chromosome with an average genome coverage depth of 475.27-fold and a G+C content of 43.92 (**Figure 3**). *B. halotolerans* LDFZ001 shares very high sequence identity (97.98%) with *B. halotolerans* F41-3. A lower sequence identity, from 82.30% to 77.80%, was also observed between *B. halotolerans* LDFZ001 and *B. subtilis* 168, *B. intestinalis* T30, *B. subtilis* ATCC 6633, *B. subtilis* FDAARGOS 606 and *B. subtilis* BJ3-2. Pairwise comparisons for the average nucleotide identity (ANI) and in silico DNA-DNA hybridization (DDH) between *B. halotolerans* LDFZ001 and these *Bacillus* family members revealed that the ANI values ranged from 87.11 to 97.98% and the DDH values ranged from 32.7 to 81.92% (**Table 1**). Based on the high ANI and DDH values between *B. halotolerans* LDFZ001 and *B. halotolerans* F41-3, both of them should belong to the same *Bacillus* species.

**Table 1:** Comparison of genome information between LDFZ001 and its six closest *Bacillus* species.

Strains	Scaffolds	Genome size (bp)	GC%	LDFZ001	
				ANI	DDH
LDFZ001	1	3965118	43.92%	100%	100%
*B. halotolerans* F41-3	1	4144458	43.76%	97.98%	81.90%
*B. intestinalis* T30	1	4031727	43.9%	87.85%	34.30%
*B. subtilis* 168	1	4215606	43.51%	87.31%	32.80%
*B. subtilis* ATCC 6633	1	4045538	43.94%	87.94%	34.40%
*B. subtilis* FDAARGOS 606	1	4045619	43.94%	87.90%	34.40%
*B. subtilis* BJ3-2	2	4200488	43.64%	87.11%	32.70%

### Genome sequence analyses of *B. halotolerans* LDFZ001

**Figure 4:**
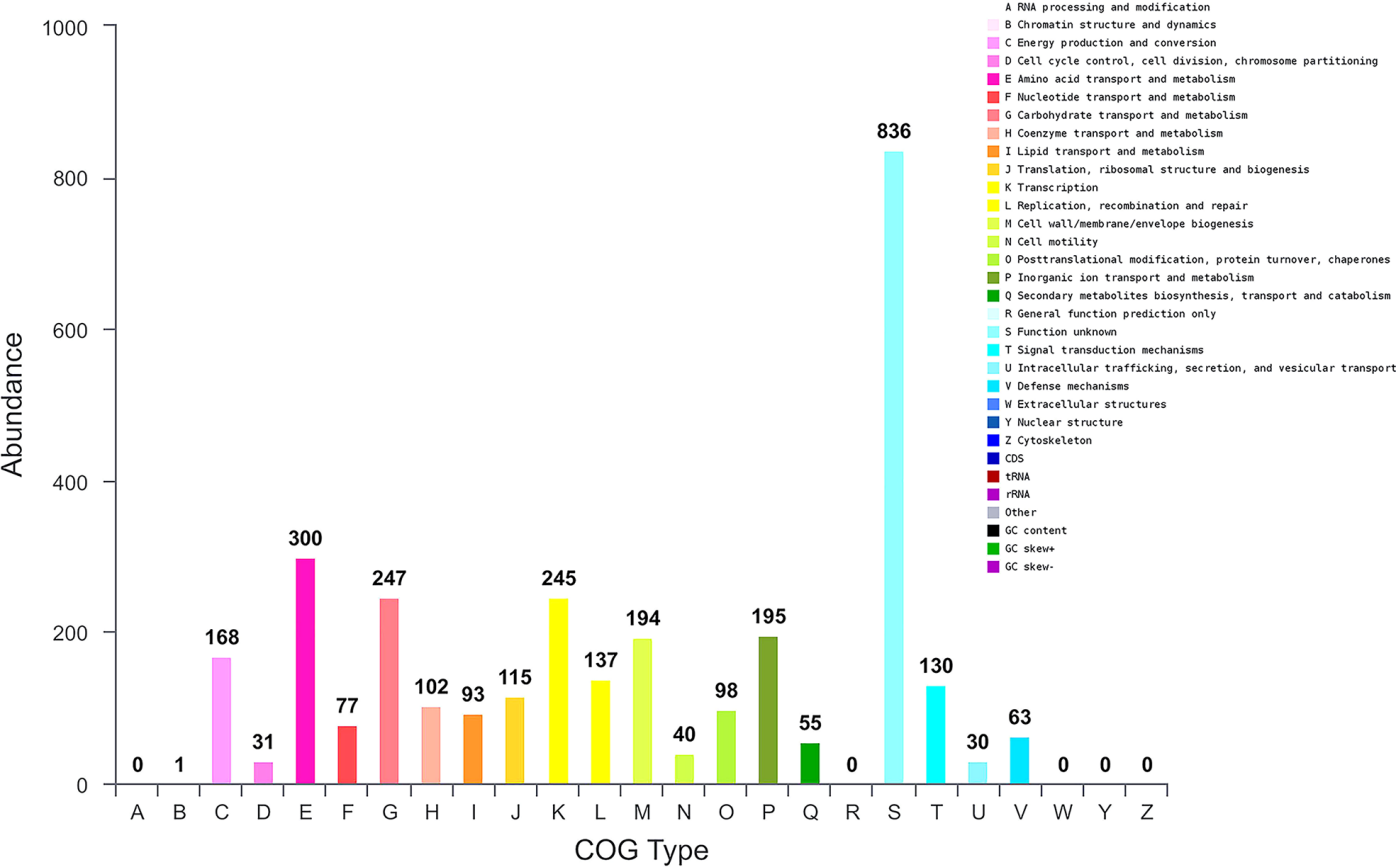
Genome sequence analysis of *B halotolerans* LDFZ001. Gene numbers involved in different pathways.

We further performed genome sequence analysis with Glimmer 3.02 and GeneMarks. The whole genome of *B. halotolerans* LDFZ001 was composed of 4,126 coding sequences (CDSs), 73 tRNAs and 24 rRNAs. The 3,500,484 bp CDSs, with an average gene length of 848 bp, accounted for 88.28%, whereas the 22 tandem repeats, with a total DNA length of 6773 bp, accounted for 0.19%, of the whole chromosome DNA (**Figure S1**). Functional classification of clusters of orthologous gene (COG) showed that the predicted genes in *B. halotolerans* LDFZ001 genome were distributed into 4 COG categories (information storage and processing, metabolism, cellular processes and signaling, and poorly characterized), including 21 COG types and 1237 metabolism, 586 cellular processes and signaling, 498 information storage and processing, and 836 functionally poorly characterized genes (**Figure 4**). The complete genome sequence of *B. halotolerans* LDFZ001, with an accession number of NZ_CP063276.1, has been deposited in the NCBI GenBank.

### A new kijanimicin biosynthesis cluster is identified in *B. halotolerans* LDFZ001

As one of the well commercialized biological control strains, *Bacillus* family can produce a diverse variety of secondary metabolites. Based on antiSMASH, a total number of 10 secondary metabolite biosynthetic gene clusters (BGCs), encoding 5 non-ribosomal peptide synthases, 2 polyketide synthase, 2 terpene synthases and 1 bacteriocin synthase, were predicted in the *B. halotolerans* LDFZ001 genome (**Table 2**). These enzymes are involved in the biosynthesis of various secondary metabolites, such as lipopeptides (surfactin and fengycin), lantipeptides (Kijanimicin and subtilosin A), dipeptide antibiotic (bacilysin), polyketides (Bacillaene), siderophores (bacillibactin) and unknown terpenes.

**Table 2:** Biosynthetic gene clusters for secondary metabolites in the genome of *B. halotolerans* LDFZ001.

Cluster ID	Type	Length (bp)	Similar Cluster	Similarity (%)	Gene Numbers
Cluster1	sactipeptide-head_to_tail	21612	Subtilosin_A_biosynthetic_gene_cluster, RiPP	100	21
Cluster2	other	41416	Bacilysin_biosynthetic_gene_cluster, nrps	100	45
Cluster3	nrps-transatpks-otherks	110082	Bacillaene_biosynthetic_gene_cluster, hybrid	100	56
Cluster4	nrps	83464	Fengycin_biosynthetic_gene_cluster, hybrid	100	47
Cluster5	nrps	49736	Bacillibactin_biosynthetic_gene_cluster, nrps	100	42
Cluster6	nrps	65394	Surfactin_biosynthetic_gene_cluster, nrps	86	49
Cluster7	lantipeptide	26864	Kijanimicin_biosynthetic_gene_cluster, polyketide	4	25
Cluster8	terpene	20776	---	–	26
Cluster9	terpene	21898	---	–	22
Cluster10	t3pks	41095	---	–	46

**Figure 5:**
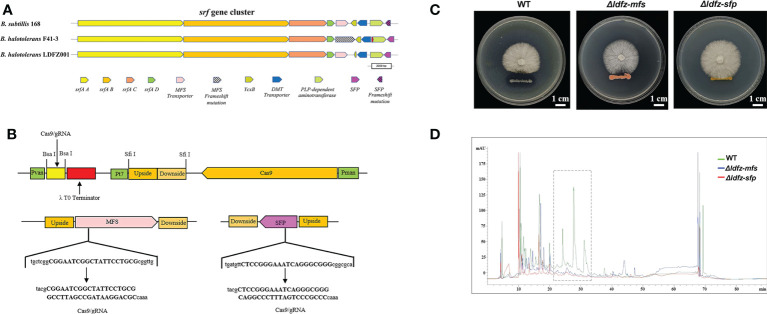
Functional analysis of SFP and MFS transporter genes. **(A)** Comparison of core genes in the srf gene clusters from *B halotolerans* LDFZ001, *B subtillis* 168 and *B halotolerans* F41-3. **(B)** A schematic map to show the gRNA sequence positions of *SFP*e and *MFS* genes. **(C)** *Δldfz*-*sfp* and *Δldfz*-*mfs* generated by respectively editing the *SFP* and *MFS* in *B halotolerans* LDFZ001 led to the loss of antifungal activity. **(D)** HPLC profile of lipopeptide extracts from WT and mutant *Δldfz*-*sfp* and *Δldfz*-*mf*.

Based on the annotation of secondary metabolite biosynthesis gene clusters, *B. halotolerans* LDFZ001 has a great potential to produce novel antibiotics. Therefore, we compared the BGCs of *B. halotolerans* LDFZ001 with other previously reported bacterial strains. Different from the bacilysin, bacillaene, fengycin, bacillibactin and subtilosin A biosynthesis clusters, which shared 100% similarity, and the surfactin biosynthesis cluster, which shared 86% similarity, a new kijanimicin biosynthesis cluster, which shared only 4% similarity, with the reported genome sequences of other bacterial strains, was identified. The schematic representation of the entire gene cluster exhibited that this novel gene cluster contained two lanthionine synthtesase C-like proteins, followed by seven ORFs encoding ATP-binding cassette (ABC) transporter proteins responsible for the translocation of a variety of metabolite compounds across membranes. Although surfactin biosynthesis cluster mainly consists of four genes, *srfAA*, *srfAB*, *srfAC* and *srfAD*, surfactin biosynthesis depends on the phosphopantetheinyl transferase and its adjacent genes. Sequence analysis showed that *B. halotolerans* LDFZ001, and the reference strains *B. subtilis* 168 and *B. halotolerans* F41-3, all possessed a complete surfactin biosynthesis cluster. However, a frameshift in *sfp* gene, downstream the surfactin biosynthesis cluster in *B. subtilis* 168, and a frameshift in the open reading frame of MFS transporter encoding gene, adjacent to *srfAD* in *B. halotolerans* F41-3, were observed. But no mutation in these two genes was observed in *B. halotolerans* LDFZ001 (**Figure 5A**).

Using CRISPR-Cas9 system, genes encoding SFP and MFS in *B. halotolerans* LDFZ001 were edited separately by a deletion of part of the gene sequences (**Figure 5B**). Two *B. halotolerans* LDFZ001 mutants, *Δldfz*-*sfp* and *Δldfz*-*mfs* were generated. Confrontation experiments showed that, unlike the wild type *B. halotolerans* LDFZ001, both *Δldfz*-*sfp* and *Δldfz*-*mfs* lost their antifungal activity against *Rhizoctonia solani Kühn* sh-1 (**Figure 5C**). Further HPLC analysis showed that the content of lipopeptide surfactin A was significantly decreased in these two mutants (**Figure 5D**).

### *B. halotolerans* LDFZ001 harbors two redundant glycoside hydrolase genes

**Figure 6:**
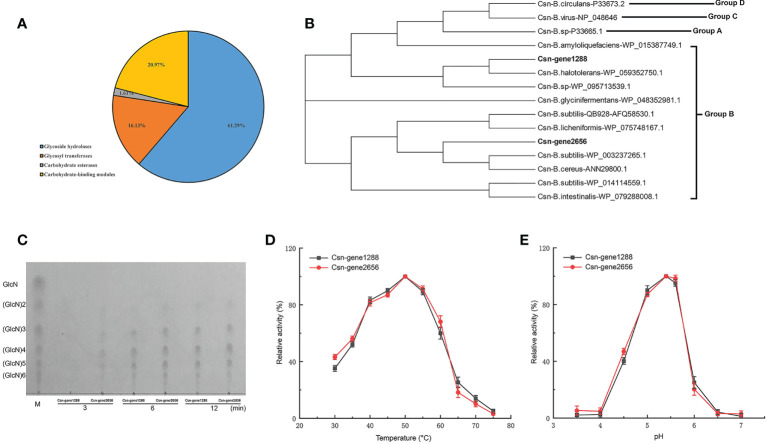
Carbohydrate-active enzyme activity analysis of the two chitosanase csn-gene1288 and csn-gene2656. **(A)** Statistical chart of annotated carbohydrate-active enzyme in *B halotolerans* LDFZ001. **(B)** Sequence alignment of csn-gene1288 and csn-gene2656 with that of other GH46 chitosanases. **(C)** Time course profiles of hydrolysis products of csn-gene1288 and csn-gene2656 enzymes on chitosan. **(D)** Effects of temperature on the enzyme activities of csn-gene1288 and csn-gene2656. **(E)** Effects of pH on the enzyme activities of csn-gene1288 and csn-gene2656.

Carbohydrate-active enzymes (CAZymes) can break down cell wall polysaccharides to trigger the death of fungal cells. We found that *B. halotolerans* LDZF001 harbored a large group of potential glycoside hydrolases, including 62 predicted CAZymes such as glycoside hydrolases, glycosyl transferases, carbohydrate esterases and carbohydrate-binding modules (**Figure 6A**). Interestingly, two members of the glycoside hydrolase GH46 family, Csn-gene1288 and Csn-gene2656, were predicted to be putative chitosanases, with a very high (90.6%) amino acid identity. Sequence alignment analysis of the deduced Csn-gene1288 and Csn-gene2656 with those of other bacterial chitosanases revealed that they were separated into two different branches (**Figure 6B**). Further enzyme activity analysis with thin-layer chromatography (TLC) showed that the hydrolysates of chitosan hydrolysed by both purified Csn-gene1288 and Csn-gene2656 were (GlcN)2, (GlcN)3, (GlcN)4, (GlcN)5 and (GlcN)6, with (GlcN) 3, (GlcN)4, and (GlcN)5 as the major products (**Figure 6C**). The catalytic properties of Csn-gene1288 and Csn-gene2656, with the optimal catalytic condition of pH5.4 at 50°C, were coincidentally similar to those of chitosanases from other *Bacillus* species (**Figures 6D, E**).

## Discussion

Biocontrol microorganisms have been well commercialized as the source of microbial pesticides. They can either be directly used or processed into pesticides. The biocontrol activities of microorganisms were largely determined by the active metabolites and hydrolases they produced (Pal et al., 2000; Nguyen et al., 2017; Chen et al., 2018). During our screening of antifungal pathogen bacterial strains, a new clone, *B. halotolerans* LDFZ001, wich could effectively inhibit the growth of fungal pathogen *Rhizoctonia solani Kühn* sh-1, was isolate (**Figures 1A, B**). Phylogenetic and whole genome sequencing analyses implied that *B. halotolerans* LDFZ001 and *B. halotolerans* F41-3 fell into the same bacterial strain category (**Figure 1C**). Although the whole genomic sequence of *B. halotolerans* LDFZ001 was smaller than that of *B. halotolerans* F41-3, they shared as high as 97.98% amino acid sequence identity (**Figure 3**; **Table 1**).

To assess the antifungal pathogen activity of *B. halotolerans* LDFZ001, we subsequently performed antagonistic activity analysis. *B. halotolerans* LDFZ001 exhibited very strong suppression, whereas the control strain *B. subtillis* 168 and *B. halotolerans* F41-3 showed nearly no inhibition, against the sheath blight pathogen strain *R. solani Kühn* sh-1 (**Figure 2A**). Further genome sequence analysis revealed that *B. halotolerans* LDFZ001 genome contained ten gene clusters related to lipopeptide and hydrolase biosynthesis (**Table 2**). Numerous studies have showed that lipopeptides and hydrolases play crucial roles in antifungal protection. We observed that in contrast to other biocontrol strains, *B. halotolerans* LDFZ001 contained two novel terpene gene clusters, one novel Type III PKS, and a new kijanimicin biosynthesis cluster. The new gene cluster, involved in the biosynthesis of kijanimicin, only shared 4% amino acid similarity with other reported genome sequences in *Bacillus* species (**Table 1**). With its specific functional mechanism, kijanimicin has provided a vital view for antibiotics research (Castiglione et al., 2008; Crowther et al., 2013). The disclosure of gene cluster for putative kijanimicin biosynthesis in the *B. halotolerans* LDFZ001 genome will provide an important gene source for the future study.

In *Bacillus* species, surfactin is a strong biological surfactant essential for the formation of mycelium (Mukherjee et al., 2006; Shen et al., 2010). Although it does not directly inhibit the growth of plant pathogenic fungi, surfactin can effectively enhance the anti-fungal activity of other lipopeptides (Kobayashi et al., 2002; Kim et al., 2017b). To date, the biosynthetic mechanism of surfactin has been deeply investigated. The *srf* gene cluster has been reported to be *sfp*-dependent. During the domestication of *B. subtilis* 168, the mutation in s*fp* gene caused the loss of its ability to produce NRPs (Wu et al., 2019). Consistently, our genome sequence analysis revealed that *B. halotolerans* LDFZ001 contained complete *sfp* gene reading frame in its *srf* gene cluster, indicating that it had ability to produce surfactin to suppress the growth of pathogenic fungi (**Figures 5A–C**). MFS transporters also play a vital role in many substances transport in both eukaryotes and prokaryotes (Saier and Paulsen, 2001; Lorca et al., 2007; Chen et al., 2008; Yen et al., 2010). We observed that, although *B. halotolerans* F41-3 has a complete s*fp* gene, a frame shift mutation was found in the open reading frame of the MFS transporter gene adjacent to *srfAD*. Therefore, the reduced antipathogen activity against *R. solani Kühn* sh-1 in *B. subtillis* 168 and *B. halotolerans* F41-3 could be due to the mutations in the *SFP* and *MFS* genes, as confirmed by the *SFP* and *MFS* gene-editing analysis with CRISPR-Cas9 system in *B. halotolerans* LDFZ001 (**Figures 5A–C**).

In prokaryotes, gene duplication only occurred among gene products in high demand, such as rRNA and histones, due to the limited sequence size (Zhang, 2003). However, to adapt the environmental changes, specific genes could be generated, giving a great contribution to the divergence of microbes (He and Zhang, 2005; Conant and Wolfe, 2008; Serres et al., 2009; Innan and Kondrashov, 2010). Although chitosanases were found to be widely distributed in both eukaryotes and prokaryotes, chitosanase gene duplication has rarely occurred (Hurst and Smith, 1998; Zhang, 2003). Two chitosanase genes in the genome of *B. halotolerans* LDFZ001 were observed, for the firstly time, in *Bacillus* species (**Figures 6B–E**). The presence of two redundant chitosanases implied that *B. halotolerans* LDFZ001 has adjusted itself to survival the variable conditions. The high enzyme activity ascribed to the duplication of chitosanase gene will also expand its utilization potential for biocontrol, chitosan production and environmental improvement of *B. halotolerans* LDFZ001.

Taken together, a new bacterial strain *B. halotolerans* LDFZ001 against sheath blight disease caused by *R. solani Kühn* sh-1 was isolated. The growth suppression ability of *B. halotolerans* LDFZ001 on *R. solani Kühn* sh-1 could be ascribed to the functional expression of *SFP* and *MFS* genes. Two redundant chitosanases, which implied the evolutionary adaption to the environment *via* gene duplication, were also verified in *B. halotolerans* LDFZ001. Our findings in this study will provide fundamental information for new candidate gene identification and bacterial strain commercialization in the future.

## Data availability statement

The datasets presented in this study can be found in online repositories. The names of the repository/repositories and accession number(s) can be found below: https://www.ncbi.nlm.nih.gov/, NZ_CP063276.1.

## Ethics statement

This article does not contain any studies with human participants or animals performed by any of the authors.

## Author contributions

ZF, MX, RZ, JY, and ZG conducted experiments. TM and YS analyzed data. JZ, ZF, and HZ wrote the manuscript. HZ polished the manuscript. All authors contributed to the article and approved the submitted version.

## Funding

This work has been jointly supported by the following grants: The National Mega Project of GMO Crops of China (2016ZX08004-002-006); The National Natural Science Foundation of China (31870576, 32071733, 31901572); The Natural Science Foundation of Shandong Province, China(ZR2019PC015, ZR2021QC140); The Cooperation Project of University and Local Enterprise in Yantai of Shandong Province (2021XDRHXMPT09); The Modern Agricultural Industry Technology System Innovation Team of Shandong Province of China (SDAIT-02-05).

## Conflict of interest

The authors declare that the research was conducted in the absence of any commercial or financial relationships that could be construed as a potential conflict of interest.

## Publisher’s note

All claims expressed in this article are solely those of the authors and do not necessarily represent those of their affiliated organizations, or those of the publisher, the editors and the reviewers. Any product that may be evaluated in this article, or claim that may be made by its manufacturer, is not guaranteed or endorsed by the publisher.

## Supplementary material

Supplementary Figure 1 A genome map of *B. halotolerans* LDFZ001.

Supplementary Table 1 Strains and plasmids used in this study.

Supplementary Table 2 Oligonucleotides used in this study.
