# Supplementary figures and images for: Molecular characterization of a novel strain of Bacillus halotolerans protecting wheat from sheath blight disease caused by Rhizoctonia solani Kühn
Source: Front Plant Sci. 2022 Oct 17;13:1019512. doi: 10.3389/fpls.2022.1019512 (PMC9618607; doi:10.3389/fpls.2022.1019512)

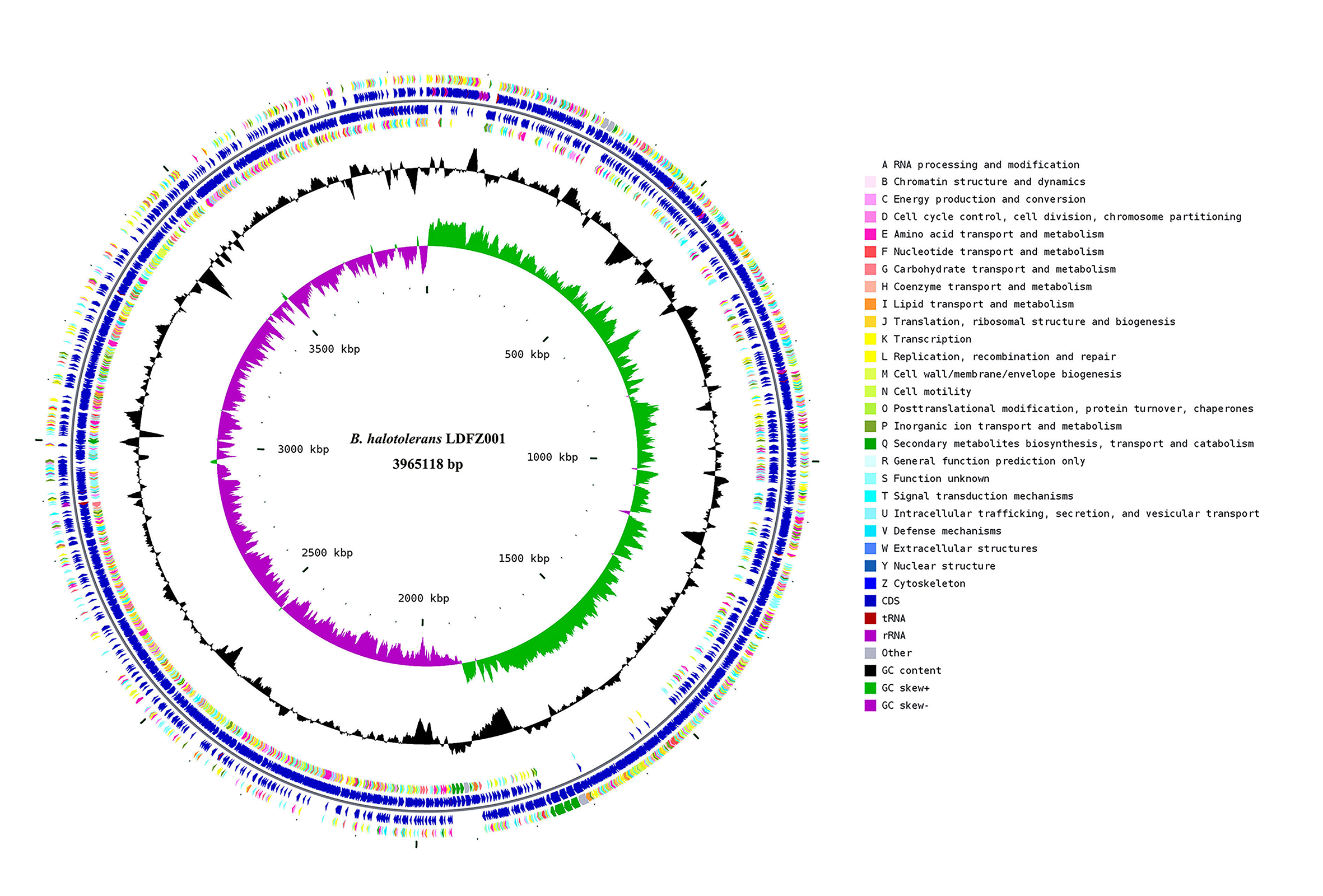

Supplement: Supplementary Figure 1 — A genome map of B. halotolerans LDFZ001. [file Image_1.tif]
