# Supplementary material for: Molecular characterization of a novel strain of Bacillus halotolerans protecting wheat from sheath blight disease caused by Rhizoctonia solani Kühn
Source: Front Plant Sci. 2022 Oct 17;13:1019512. doi: 10.3389/fpls.2022.1019512 (PMC9618607; doi:10.3389/fpls.2022.1019512)
Supplement: Supplementary Table 1 — Strains and plasmids used in this study. [file Table_1.doc]

**SupplementaryTable 1** Strains and plasmids used in this study

| Strains/ Plasmids | Description | Reference/source |
| --- | --- | --- |
| LDFZ001 | Wild type *B. halotolerans* isolated from the coastal zone of Yantai | This study |
| *B. subtillis* 168 | control strain | BioSciBio |
| *B. halotolerans* FF41-3 | control strain | BioSciBio |
| *R. solani Kühn* Sh-1 | pathogenic microbe | from Dr Sheng |
| Δldfz-sfp | LDFZ001, Δsfp | This study |
| Δldfz-mfs | LDFZ001, Δmfs | This study |
| pEASY-T1 | ApR, KanR, pUC origin, 3.8-kb, TA cloning vector | Transgen |
| pET28a (+) | pET28a(+), KanR, T7 promoter, pBR322 origin, f1 origin | Invitrogen |
| pJOE8999 | for editing of genes by CRISPR-Cas 9 system | (Altenbuchner 2016) |
| pJOEsfp | For editing of gene SFP | This study |
| pJOEmft | For editing of gene MFT transporter | This study |
| pET28a-Csn1288 | For expression of Gene Csn1288 | This study |
| pET28a-Csn2656 | For expression of Gene Csn2656 | This study |

**Supplementary Table 2** Oligonucleotides used in this study

| Name | Sequence | Purpose |
| --- | --- | --- |
| MFSgRNA-F | ggaaatggacgcagcagtcag**gttttagagctagaaatagcaag** | Insert MFSgRNA into pJOE8999 |
| MFSgRNA-R | cctgactgctgcgtccatttc**cgtaggtacattttactcaattc** |
| MFSup-F | **ctatagggtcgacggccaacg**atgaactcattcagaaattcc | PCR of fragment of MFS upside |
| MFSup-R | **aaattattggcccgggcc**tcgcattcggcgactgcgggagcg |
| MFSdown-F | **aatgcgaggcccgggcc**aataatttctttatccatcaaatcg | PCR of fragment of MFS downside |
| MFSdown-R | **cttaatctagaaaggcc**ttatttacccctcaacactctcttcg |
| SFPgRNA-F | aaccccgccctgatttcccggag**cgtaggtacattttactcaattc** | Insert SFPgRNA into pJOE8999 |
| SFPgRNA-R | acgctccgggaaatcagggcggg**gttttagagctagaaatagcaag** |
| SFPup-F | **ctatagggtcgacggccaacg**tgtgaaagcaactccgcctatac | PCR of fragment of SFP upside |
| SFPup-R | **acgagtattggcccgggcc**tcgggctttattcagctggtactgc |
| SFPdown-F | **aagcccgaggcccgggcc**aatactcgtacgaagcgcttttataat | PCR of fragment of SFP downside |
| SFPdown-R | **cttaatctagaaaggcc**ttattacccatctggtgcttcctgag |
| Csn1288-F | **ctggtgccgcgcggcagccat**atgaaaatcagtttggagaa | construct of pET28a-Csn1288 |
| Csn1288-R | **gtggtggtggtggtgctcgag**ttatttgattacgaaatcaccg |
| Csn2656-F | **ctggtgccgcgcggcagccat**atgaaaatcagtttgaagaa | construct of pET28a-Csn2656 |
| Csn2656-R | **gtggtggtggtggtgctcgag**ttaattgattacaaaattacc |

**Bold represents** **homologous recombination sequences.**
